# Associations Between Obesity‐Related Comorbidities and Weight Loss After Bariatric Surgery: A Retrospective Analysis

**DOI:** 10.1155/jobe/8878737

**Published:** 2026-07-18

**Authors:** Urszula Stepaniak, Urszula Ambroży, Magdalena Kozela, Izabela Karpińska, Piotr Major, Maciej Matyja

**Affiliations:** ^1^ Department of Epidemiology and Population Studies, Institute of Public Health, Jagiellonian University Medical College, Skawinska St. 8, 31-066, Krakow, Poland, cm-uj.krakow.pl; ^2^ 2nd Department of General Surgery, Jagiellonian University Medical College, Jakubowskiego St. 2, 30-688, Krakow, Poland, cm-uj.krakow.pl

**Keywords:** bariatric surgery, body weight reduction, comorbidity, effectiveness of bariatric treatment, metabolic syndrome, obesity

## Abstract

**Objective:**

The aim of the study was to describe the prevalence of comorbidities in patients with severe obesity in Poland and to assess the relationship between the diagnosis of chronic diseases and total weight lost after bariatric surgery in 1 year observation.

**Patients and Methods:**

The current study is a retrospective analysis that included medical records of 601 patients with data on body weight before and 1 year after bariatric surgery. The total weight loss was calculated as the difference between the initial body weight and body weight after 1 year, divided by the initial body weight, multiplied by 100. The total weight loss was calculated separately for body weight at admission for the surgery (%WL) and maximum recorded body weight (%maxWL). Data on history of all recorded in medical documentation chronic diseases were used. The associations between the presence of chronic diseases at baseline and total weight loss were assessed using multivariable linear regression.

**Results:**

Hypertension was the most common chronic disease (71.2%). Patients with hypertension, metabolic syndrome, and chronic obstructive pulmonary disease (COPD) had significantly higher %WL and %maxWL, while those with osteoarthritis and gastroesophageal reflux disease (GERD) had lower %WL and %maxWL. After adjusting for age, sex, and initial body mass index (BMI), the presence of metabolic syndrome and COPD was positively associated with %WL (respectively, beta = 2.405; 95% CI: 0.195–4.615 and beta = 6.522; 95% CI: 1.094–11.951) and with %maxWL (respectively, beta = 2.389; 95% CI: 0.249–4.528 and beta = 6.281; 95% CI: 1.038–11.524). All other diseases and total number of comorbidities were not related to total weight loss.

**Conclusion:**

Among patients undergoing bariatric surgery, obesity‐related comorbidities were highly prevalent at baseline and did not adversely affect postoperative weight loss. Specifically, the presence of metabolic syndrome and COPD was independently associated with a greater 1‐year total weight loss.

## 1. Introduction

Due to the high prevalence of obesity and its serious health and social and economic consequences, effective treatment for this condition is an important current public health problem. Bariatric procedures are methods of treating morbid obesity with proven effectiveness [1, 2]. Surgeries are no longer regarded as a treatment of excess body weight alone, but even more important are beneficial metabolic effects [3, 4]. Bariatric procedures are suggested to be more effective than nonsurgical methods for weight loss achievement [5]. There is growing evidence proving the effectiveness of bariatric surgery on the treatment of obesity‐related chronic diseases [6–14]. Apart from beneficial metabolic changes, in patients with chronic obstructive pulmonary disease (COPD) and obesity, the risk of acute exacerbation of COPD significantly decreased after bariatric surgery [15].

There are different factors influencing optimal clinical response for primary procedures. Besides demographic, anthropometric, and social factors or behavioral problems, the presence of other chronic diseases may have an effect. However, the impact of the presence of other diseases on postoperative weight loss considering research is not univocal. Regarding the most common chronic diseases, the presence of diabetes mellitus was related with less weight loss [16–23] or had no effect [24–26]. However, preoperative insulin use was related with greater postoperative weight loss in long term [27]. Diagnosis of hypertension was related with poorer weight loss in some studies [23, 24, 26, 28], but not in all [16, 17, 19, 20, 25, 29]. Impact of diseases occurring less frequently in bariatric patients was much less examined. Very few studies assessed the impact of metabolic syndrome [22], gastroesophageal reflux disease (GERD) [20, 29], cardiovascular events [20, 24, 29], or osteoarthritis [16, 20, 24, 28] on weight loss after bariatric treatment, and we did not find studies on the impact of COPD. Influence of the total number of comorbidities on weight loss was assessed in few studies but results were inconclusive [20, 27, 28].

Poland is the postsocialist country, which in the last decades underwent profound socioeconomic changes [30]. Despite many beneficial changes observed in the Polish society, the East–West health gap is still significant. Life expectancy at birth in Poland is lower than average in EU countries, smoking and obesity rates are above the EU average, and other lifestyle and socioeconomic circumstances also differ from those in Western countries, where research on weight loss after bariatric treatment was mainly conducted [31, 32]. The first bariatric procedures in Poland were performed in the 1970s, and laparoscopy was introduced in the late 1990s [33]. A steep increase in the number of bariatric procedures in Poland has been observed in recent years: from 176 in 2007 to almost 2000 in 2016 years [34]. The impact of the presence of chronic diseases on postoperative weight loss in the societies with poorer health, which Poland represents, has not been published earlier. The aim of this study was to describe the prevalence of comorbidities in patients with severe obesity in Poland and to assess the relationship between the diagnosis of chronic diseases and total weight loss after bariatric surgery in a 1‐year observation.

## 2. Material and Methods

### 2.1. Studied Group

Data were retrospectively collected from the medical records of all patients who were admitted between January 1, 2009 and December 31, 2021 in the 2^nd^ Department of General Surgery Jagiellonian University Medical College in order to receive surgical treatment of obesity. Public reimbursement for bariatric surgery in Poland was available to all patients aged ≥ 18 years with severe obesity defined by BMI (BMI ≥ 40 kg/m^2^ or BMI 35–39.9 kg/m^2^ with obesity‐related comorbidities) who, after appropriate medical evaluation, received a referral from their primary care physician or specialist to a general surgery clinic. Between 2009 and 2021, there were a total number of 1451 records of patients that underwent bariatric surgery. Subsequently, 850 patients were excluded due to missing body weight measurements in their medical records within 1 year following bariatric surgery. Finally, the studied group consisted of 601 patients for whom information about body weight at follow‐up (1 year after bariatric surgery) was recorded. The study was planned and conducted in accordance with the Declaration of Helsinki. The Committee for Ethics of Scientific Research at the Jagiellonian University Medical College approved the study (dec. no. 118.0043.1.479.2024).

### 2.2. Data

The data were extracted from the hospital’s medical records using procedure codes related to bariatric surgery. Relevant variables required to address the study objectives were compiled into an Excel file. Based on medical records, we retrospectively obtained information, which included sex, age, maximum body weight, body weight and body height on admission for surgery, body weight 1 year after surgery, and type of procedure (Laparoscopic Roux‐en‐Y gastric bypass (RYGB) or laparoscopic sleeve gastrectomy (SG)). We used data on history of all recorded in medical documentation chronic diseases, which included doctor’s diagnosis of diabetes, hypertension, metabolic syndrome, heart failure of any etiology, coronary artery disease, COPD, asthma, varicose veins of the lower limbs, osteoarthritis, GERD, hepatic steatosis, and periodic back and joint pain. The medical documentation contained complete information on all diagnoses for each patient. All comorbidities were reviewed and taken into account by our team during the decision‐making process prior to bariatric surgery. The surgeon made a conclusive decision based on the results of consultations performed by a multidisciplinary team. The team at our department consists of dieticians, psychologists, cardiologists, pulmonologists, anesthesiologists, and coordinating nurse. All other consultations (e.g. psychiatrists, ENT specialists) were performed if necessary.

The data were checked for completeness and accuracy. Continuous variables, such as body weight, were inspected for implausible values and verified and corrected as necessary. Information on chronic diseases was coded dichotomously (present/absent). The total weight loss was calculated as the difference between the initial body weight and body weight after 1 year, divided by the initial body weight, multiplied by 100%. The total weight loss was calculated separately for maximum recorded body weight (%maxWL) and body weight at admission for the surgery (%WL). The excess weight loss was calculated as the difference between the initial body weight and body weight after 1 year divided by the difference between the initial body weight and ideal body weight, multiplied by 100. The ideal body weight was calculated separately for male and female patients based on the Broca formula. For males, the ideal body weight was calculated as 90% of (height in centimeters minus 100), and for females, it was 85% of (height in centimeters minus 100). The excess weight lost was calculated separately for the maximum recorded body weight (% max EWL) and body weight at admission for the surgery (%EWL). BMI on admission was calculated as one’s weight in kilograms divided by the square of height in meters. The BMI was calculated separately for the maximum recorded body weight and body weight at admission for the surgery. The excess BMI loss was calculated by dividing the difference between the initial BMI and BMI 1 year after surgery by the difference between the initial BMI and a “normal” target BMI below 25 kg/m^2^, multiplied by 100 (%EBMIL).

### 2.3. Statistical Analysis

Descriptive statistics were presented as means and standard deviations or medians and Q1–Q3, as appropriate. To test the normality of the distribution of continuous variables, the Shapiro–Wilk test was used. Categorical variables were described using numbers of patients (N) and percentages (%) and compared using the Chi2 (Chi‐square) test. To investigate the differences in %WL and %maxWL between patients with and without chronic diseases, the t‐Student test was used. For the results that were significantly different, multivariable linear regression was performed to investigate the association between the history of chronic diseases and %WL and %maxWL.

In the linear regression analysis, the dependent variable was the total weight loss (%WL or %maxWL); the independent variables were coded as follows: the presence of each comorbidity was treated as a binary variable (yes/no), while the total number of comorbidities was analyzed as a categorical variable with four groups (coded as no comorbidities, one comorbidity, 2–3 comorbidities (reference), and ≥ 4 comorbidities). Firstly, in univariable models, the associations between the total weight loss and each chronic disease and the number of comorbidities were assessed (Model 1). Further, multivariable analysis was performed including age and sex as covariates (Model 2). Additional adjustment for initial BMI was made (Model 3). Subsequent models are nested and were constructed stepwise, illustrating the potential impact of blocks of covariates on the association of interest. Interaction effects between chronic conditions were not formally tested, as the primary focus of the analysis referred to the independent associations of each condition and the available sample size limited the ability to assess higher‐order interactions. The analysis was performed using Statistica version 13 from TIBCO Software Inc. *p*‐values < 0.05 were accepted as statistically significant.

## 3. Results

There were 601 patients (67.05% of women and 32.95% of men) who had data on the initial body weight and body weight 1 year after surgery. The mean age of participants was 42.0 years (Table 1). The youngest patient was 18.0 years old, and the oldest was 68.0 years old. The mean height of patients was 169.0 cm. The mean body weight at admission to bariatric surgery was 130 kg, and the mean maximum body weight was 135.0 kg. The mean BMI at admission for the surgery was 45.2 kg/m^2^, and the mean maximum BMI was 46.7 kg/m^2^. The majority of the patients underwent SG (63.2%). The mean number of comorbidities in the studied group was 3, and only 13.3% patients had no comorbidities. Additionally, comparison of demographic and anthropometric traits of 601 participants with the remaining patients for whom no data on body weight after 1 year were available is presented in the Supporting table 1. There were no statistically significant differences between patients included in the analysis and patients with no data on body weight 1 year after surgery in terms of mean age, sex distribution, mean maximum body weight, or the majority of the evaluated comorbidities (COPD, asthma, osteoarthritis, heart failure of any etiology, varicose veins of the lower limbs, GERD, hepatic steatosis, and periodic back and joint pain).

**TABLE 1:** Characteristics of total study group and within groups according to weight change.

Characteristics	Total *N* = 601	Weight gain and no change in body weight after surgery *N* = 54	Weight loss after surgery *N* = 547	*p* value
Sex				
Male, *n* (%)	198 (32.95)	8 (14.8)	190 (34.7)	0.003^∗^
Female, *n* (%)	403 (67.05)	46 (85.2)	357 (65.3)	
Age, median (Q1–Q3) years	42.0 (35.0–51.0)	41.0 (34.0–51.0)	42.0 (35.0–51.0)	0.524
Height, median (Q1–Q3) cm	169.0 (164.0–176.0)	166.0 (162.0–170.0)	170.0 (164.0–176.0)	< 0.001^∗^
Weight before bariatric surgery, median (Q1–Q3) kg	130.0 (116.5–146.0)	109.5 (100.0–118.0)	132.0 (120.0–148.0)	< 0.001^∗^
BMI before bariatric surgery, median (Q1–Q3) kg/m^2^	45.2 (41.5–49.6)	39.1 (37.1–42.6)	45.5 (41.9–50.5)	< 0.001^∗^
Weight max, median (Q1–Q3) kg	135.0 (120.0–152.0)	118.0 (108.0–126.0)	137.0 (124.0–155.0)	< 0.001^∗^
maxBMI, median (Q1–Q3) kg/m^2^	46.7 (43.2–51.6)	42.9 (39.0–46.0)	48.5 (43.9–52.4)	< 0.001^∗^
Type of bariatric surgery				
RYGB, *n* (%)	221 (36.8)	23 (42.6)	198 (36.2)	0.353
SG, *n* (%)	380 (63.2)	31 (57.4)	349 (63.8)	
Number of comorbidities				
0, *n* (%)	80 (13.3)	8 (14.8)	72 (13.2)	0.854
1, *n* (%)	92 (15.3)	10 (18.5)	82 (15.0)	
2–3, *n* (%)	261 (43.4)	21 (38.9)	240 (43.9)	
4 +, *n* (%)	168 (28.0)	15 (27.8)	153 (28.0)	
Number of comorbidities, median (Q1–Q3)	3.0 (1.0–4.0)	3.0 (1.0–4.0)	3.0 (1.0–4.0)	0.822

*Note: N*, number of patients; Q1–Q3, quartile 1‐quartile 4. RYGB, laparoscopic Roux‐en‐Y gastric bypass; SG, laparoscopic sleeve gastrectomy.

Abbreviation: BMI, body mass index.

^∗^ *p* value < 0.05.

In the studied group, 547 patients (91.0%) lost weight in a 1‐year follow‐up. The remaining 5 patients (0.8%) did not change body weight or 49 patients (8.2%) gained body weight after a 1‐year follow‐up. Characteristics of the studied group by postoperative weight change are presented in Table 1. There were no statistical differences regarding the mean age, the type of bariatric surgery, and number of comorbidities. In the group that lost weight, the percent of men as well as mean preoperative body weight was higher.

Table 2 shows the prevalence of chronic diseases overall and by body weight change. Hypertension was the most prevalent chronic disease among study participants (71.2%). Metabolic syndrome was recorded in 59.7% patients, diabetes in 33.6%, varicose veins of the lower limbs in 20.8%, osteoarthritis in 18.6%, hepatic steatosis in 11.3%, and GERD in 11.2%. Less frequent were diagnoses of heart failure (8.5%), periodic back and joint pain (8.7%), coronary artery disease (6.8%), asthma (6.8%), and COPD (3.5%). There were no statistically significant differences between the prevalence of chronic diseases in patients that gained weight or had no change in body weight after surgery compared to patients that lost weight after surgery.

**TABLE 2:** Percent of chronic diseases in total group and according to weight change groups.

Diseases	Total *N* = 601	Weight gain and no change in body weight after surgery *N* = 54	Weight loss after surgery *N* = 547	*p* value
Diabetes, *n* (%)	202 (33.6)	19 (35.2)	183 (33.5)	0.798
Hypertension, *n* (%)	427 (71.2)	34 (63.0)	393 (72.0)	0.163
Metabolic syndrome, *n* (%)	359 (59.7)	34 (63.0)	325 (59.4)	0.613
Heart failure of any etiology, *n* (%)	51 (8.5)	3 (5.6)	48 (8.8)	0.418
Coronary artery disease, *n* (%)	41 (6.8)	3 (5.6)	38 (7.0)	0.699
COPD, *n* (%)	21 (3.5)	0 (0.0)	21 (3.8)	
Asthma, *n* (%)	41 (6.8)	3 (5.6)	38 (7.0)	0.699
Varicose veins of the lower limbs, *n* (%)	125 (20.8)	11 (20.4)	114 (20.8)	0.935
Osteoarthritis, *n* (%)	112 (18.6)	13 (24.1)	99 (18.10)	0.282
GERD, *n* (%)	67 (11.2)	7 (13.0)	60 (11.0)	0.657
Hepatic steatosis, *n* (%)	68 (11.3)	5 (9.3)	63 (11.5)	0.617
Periodic back and joint pain, *n* (%)	52 (8.7)	6 (11.1)	46 (8.4)	0.501

Abbreviations: COPD, chronic obstructive pulmonary disease; GERD, gastroesophageal reflux disease.

In the group that lost weight in a 1‐year observation, %WL and %maxWL were normally distributed, with mean values of 30.79% (SD 14.84%) and 33.50% (SD 14.42%), respectively (Supporting table 2). The distributions of the remaining variables were as follows: mean %EWL was 56.5% (SD 26.44%) and mean %EBMIL was 67.19% (SD 30.28%). The indicators calculated for maximum recorded body weight were for about 3% points higher than calculated for body weight at admission.

As presented in Table 3, patients with hypertension, metabolic syndrome, and COPD had significantly higher %WL compared to patients without these chronic conditions. But in those with diagnosis of osteoarthritis and GERD, significantly lower %WL was recorded compared to patients without these chronic conditions.

**TABLE 3:** Association between the presence of chronic diseases and total weight loss in a 1‐year observation.

Chronic diseases		%WL^a^		*p* value (*t*‐student)	%maxWL^b^		*p* value (*t*‐student)
		Mean (%)	SD		Mean (%)	SD	
Diabetes	yes	32.2	14.52	0.114	35.3	14.03	0.036^∗^
	no	30.1	14.96		32.6	14.55	

Hypertension	yes	31.8	14.96	0.010^∗^	34.5	14.65	0.005^∗^
	no	28.1	14.25		30.7	13.51	

Metabolic syndrome	yes	32.1	15.00	0.014^∗^	34.9	14.62	0.006^∗^
	no	28.9	14.42		31.5	13.91	

Heart failure of any etiology	yes	30.6	14.77	0.915	34.2	15.12	0.716
	no	30.8	14.77		33.4	14.37	

Coronary artery disease	yes	31.3	16.83	0.829	35.1	15.80	0.475
	no	30.8	14.69		33.4	14.32	

COPD	yes	39.7	14.56	0.005^∗^	42.2	13.79	0.005^∗^
	no	30.4	14.75		33.2	14.35	

Asthma	yes	31.4	17.61	0.793	34.3	17.06	0.717
	no	30.7	14.63		33.4	14.22	

Varicose veins of the lower limbs	yes	30.7	14.64	0.955	33.8	13.81	0.832
	no	30.8	14.90		33.4	14.60	

Osteoarthritis	yes	27.7	15.40	0.021^∗^	29.9	15.19	0.006^∗^
	no	31.5	14.64		34.3	14.14	

GERD	yes	27.0	13.94	0.035^∗^	29.6	13.86	0.027^∗^
	no	31.3	14.89		34.0	14.43	

Hepatic steatosis	yes	31.7	14.80	0.594	34.5	14.19	0.570
	no	30.7	14.85		33.4	14.46	

Periodic back and joint pain	yes	32.4	13.91	0.436	35.1	12.84	0.440
	no	30.6	14.92		33.4	14.56	

Abbreviations: COPD, chronic obstructive pulmonary disease; GERD, gastroesophageal reflux disease; SD, standard deviation.

^a^%WL‐percent of the total weight loss calculated for body weight at admission for the surgery.

^b^%maxWL‐percent of the total weight loss calculated for maximum recorded body weight.

^∗^ *p* value < 0.05.

Similarly to %WL, significantly greater %maxWL was found among patients with hypertension, metabolic syndrome, COPD, and additionally among diabetes patients; but lower %maxWL was observed in those with osteoarthritis or GERD (Table 3).

The results of multivariable linear regression are presented in Table 4 for %WL. After adjustment for age, sex, and initial BMI, there were significant positive associations between %WL and preoperative diagnosis of metabolic syndrome (*b* = 2.405, 95% CI: 0.195–4.615) and COPD (*b* = 6.522, 95% CI: 1.094–11.951). Patients with metabolic syndrome had 2.4% greater weight loss and with COPD had 6.5% greater %WL. Associations between %WL and hypertension, osteoarthritis, GERD, and total number of comorbidities were explained by the differences in initial BMI.

**TABLE 4:** Association between chronic diseases and total weight loss calculated for body weight at admission for the surgery (%WL)—results of multivariable linear regression.

Diseases	Beta	Standard error	95% CI		*p* value
			Lower	Upper	
Metabolic syndrome					
Model 1^a^	3.177	1.286	0.652	5.703	0.014^∗^
Model 2^b^	3.686	1.259	1.214	6.158	0.004^∗^
Model 3^c^	2.405	1.125	0.195	4.615	0.033^∗^
Hypertension					
Model 1^a^	3.641	1.407	0.877	6.404	0.010^∗^
Model 2^b^	2.929	1.468	0.046	5.812	0.046^∗^
Model 3^c^	1.589	1.309	−0.982	4.160	0.225
COPD					
Model 1^a^	9.268	3.280	2.824	15.712	0.005^∗^
Model 2^b^	7.809	3.112	1.695	13.923	0.012^∗^
Model 3^c^	6.522	2.763	1.094	11.951	0.019^∗^
Osteoarthritis					
Model 1^a^	−3.788	1.641	−7.012	−0.565	0.021^∗^
Model 2^b^	−2.539	1.594	−5.671	0.593	0.112
Model 3^c^	−2.114	1.414	−4.893	0.664	0.135
GERD					
Model 1^a^	−4.288	2.023	−8.262	−0.313	0.035^∗^
Model 2^b^	−3.624	1.904	−7.365	0.117	0.058
Model 3^c^	−2.179	1.696	−5.510	1.152	0.199
Number of comorbidities (ref. 2 & 3)					
0					
Model 1^a^	−4.293	1.986	−8.194	−0.392	0.031^∗^
Model 2^b^	−4.350	1.940	−8.161	−0.540	0.025^∗^
Model 3^c^	−3.100	1.728	−6.494	0.293	0.073
1					
Model 1^a^	−3.856	1.891	−7.569	−0.142	0.042^∗^
Model 2^b^	−4.001	1.785	−7.508	−0.494	0.025^∗^
Model 3^c^	−2.803	1.590	−5.927	0.320	0.079
4+					
Model 1^a^	−1.719	1.529	−4.722	1.285	0.261
Model 2^b^	−1.210	1.502	−4.159	1.740	0.421
Model 3^c^	−1.102	1.335	−3.725	1.520	0.409

Abbreviations: COPD, chronic obstructive pulmonary disease; GERD, gastroesophageal reflux disease.

^a^Model 1—crude.

^b^Model 2—adjusted to age and sex.

^c^Model 3—adjusted to age, sex, and initial BMI.

^∗^ *p* value < 0.05.

In Table 5, the results of multivariable linear regression for %maxWL were presented. The observed positive association between %max WL and diabetes was insignificant after adjustment for initial BMI. The remaining results were consistent with the results for %WL. Additional adjustment for the type of surgical procedure did not alter the results (Supporting Tables 3 and 4).

**TABLE 5:** Association between selected chronic diseases and total weight loss calculated for maximum recorded body weight (%maxWL)—results of multivariable linear regression.

Diseases	Beta	Standard error	95% CI		*p* value
			Lower	Upper	
Diabetes					
Model 1^a^	2.740	1.303	0.181	5.300	0.036^∗^
Model 2^b^	3.078	1.290	0.543	5.612	0.017^∗^
Model 3^c^	2.068	1.145	−0.180	4.320	0.071
Metabolic syndrome					
Model 1^a^	3.423	1.248	0.971	5.876	0.006^∗^
Model 2^b^	3.949	1.218	1.555	6.342	0.001^∗^
Model 3^c^	2.389	1.089	0.249	4.528	0.029^∗^
Hypertension					
Model 1^a^	3.810	1.366	1.126	6.494	0.005^∗^
Model 2^b^	3.143	1.422	0.351	5.936	0.027^∗^
Model 3^c^	1.527	1.266	−0.961	4.014	0.228
COPD					
Model 1^a^	9.064	3.189	2.799	15.328	0.005^∗^
Model 2^b^	7.614	3.018	1.685	13.543	0.012^∗^
Model 3^c^	6.281	2.669	1.038	11.524	0.019^∗^
Osteoarthritis					
Model 1^a^	−4.406	1.592	−7.533	−1.279	0.006^∗^
Model 2^b^	−3.214	1.544	−6.246	−0.182	0.038^∗^
Model 3^c^	−2.258	1.367	−4.943	0.428	0.099
GERD					
Model 1^a^	−4.371	1.966	−8.233	−0.508	0.027^∗^
Model 2^b^	−3.714	1.846	−7.340	−0.088	0.045^∗^
Model 3^c^	−2.186	1.638	−5.404	1.032	0.183
Number of comorbidities (ref. 2 & 3)					
0					
Model 1^a^	−4.877	1.927	−8.663	−1.091	0.012^∗^
Model 2^b^	−5.047	1.877	−8.733	−1.360	0.007^∗^
Model 3^c^	−3.213	1.671	−6.496	0.070	0.055
1					
Model 1^a^	−4.056	1.835	−7.660	−0.452	0.027^∗^
Model 2^b^	−4.240	1.727	−7.633	−0.848	0.014^∗^
Model 3^c^	−2.885	1.536	−5.902	0.132	0.060
4+					
Model 1^a^	−1.498	1.484	−4.413	1.417	0.313
Model 2^b^	−0.901	1.453	−3.755	1.953	0.535
Model 3^c^	−0.956	1.289	−3.488	1.575	0.458

Abbreviations: COPD, chronic obstructive pulmonary disease; GERD, gastroesophageal reflux disease.

^a^Model 1 ‐crude.

^b^Model 2 ‐adjusted to age and sex.

^c^Model 3 ‐adjusted to age, sex, and initial maxBMI.

^∗^ *p* value < 0.05.

**FIGURE 1:**
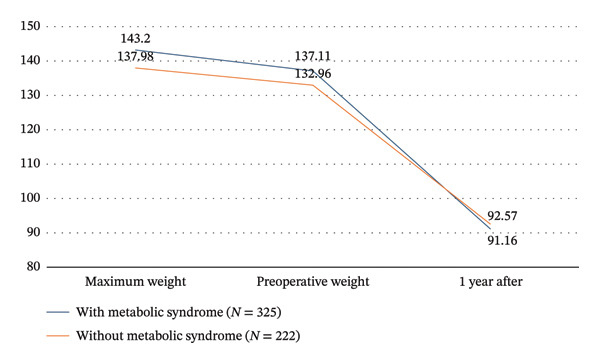
Change in average recorded weight (kg) among patients with and without metabolic syndrome that lost weight.

**FIGURE 2:**
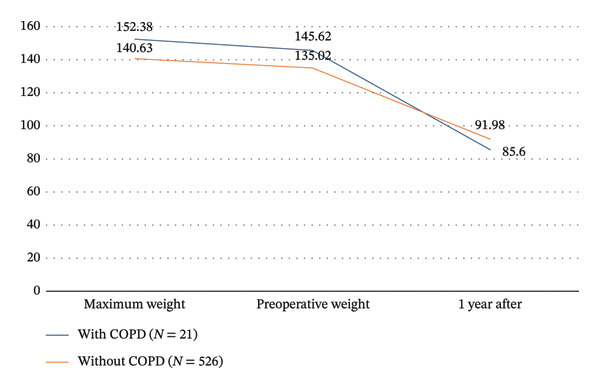
Change in average recorded weight (kg) among patients with and without chronic obstructive pulmonary disease (COPD) that lost weight.

Figures 1 and 2 show the mean maximum body weight, body weight at admission, and body weight at the end of 1‐year follow‐up for patients with and without metabolic syndrome as well as for patients with and without COPD. The significant difference in %WL in individuals with metabolic syndrome is largely due to the fact that they had a higher initial body weight (Figure 1). Patients with COPD also had a higher baseline body weight, but they also had a lower body weight at 1‐year follow‐up (Figure 2).

## 4. Discussion

Metabolic surgery is known to achieve and sustain improvements in metabolic dysfunction secondary to obesity. Successful bariatric surgery is dependent not only on demographic, psychological, and behavioral factors but also on clinical condition. Our study shows that only 13% of patients with severe obesity have no comorbidities, and the average number of comorbidities was three. Additionally, among people who lost weight after bariatric surgery, those who were diagnosed with metabolic syndrome or COPD lost weight more effectively in 1‐year follow‐up, compared to patients without these conditions. Our observations are statistically significant after adjustment for gender, age, and baseline BMI.

Impact of preoperative diagnosis of metabolic syndrome on postoperative weight loss in our study was beneficial. Metabolic syndrome is the co‐occurrence of several disorders; therefore, due to the increased risk in these patients, they might receive more attentive care to better monitor their condition. As a consequence, their weight loss might be better supervised both before the surgery and during follow‐up. In other studies, patients with a greater percentage of missed preoperative appointments had a lower postoperative %EWL 1 year after gastric banding; however, this relationship was not confirmed for all types of treatments [35]. In addition to being better monitored, patients with chronic diseases could be better motivated to lose weight as they are also expecting improvement in the metabolic condition. Even in lifestyle interventions, it was confirmed that patient‐specific factors, including cardiometabolic comorbidities, were significantly associated with more frequent weight loss success [36]. Very few studies assessed the impact of diagnosis of metabolic syndrome on the postoperative body weight change. Contrary to our results, the occurrence of metabolic syndrome was associated with the suboptimal clinical response in the study of 771 patients after laparoscopic gastric bypass in Italy [22]. According to Lak et al., metabolic syndrome increased the risk of mortality and morbidity after bariatric surgery and such comorbidities brought additional risk of undergoing bariatric surgery [37].

In our study, to the best of our knowledge, the impact of COPD on weight loss after bariatric surgery was assessed for the first time, and diagnosis of COPD was related with higher postoperative weight loss. Possible explanation for this finding, similarly, like in the metabolic syndrome, is a greater interaction of patients with COPD with the healthcare system, leading to better adherence and ultimately better weight loss. Patients with COPD receiving treatment are supported with continuous positive airway pressure (CPAP), but their overall course of treatment is the same as that of other patients.

Metabolic and other disorders are common in patients with severe obesity, and it might seem that the burden of chronic diseases may contribute to a poorer response to bariatric treatment. However, in our study, the presence of the remaining diseases (diabetes, hypertension, cardiovascular disorders, asthma, GERD, osteoarthritis, hepatic steatosis, joint pain) or total number of comorbidities were not associated with postoperative weight loss, which is in line with another research [16, 17, 19, 20, 24–26, 28, 29]. Similarly to our findings, diabetes was not associated with postoperative weight loss in studies conducted in Mexico [24], the USA [25], and Chile [26]. Likewise, the lack of association between hypertension and weight loss observed in our study aligns with reports from the USA [16, 20, 25, 29] and Brazil [17, 18]. However, other studies [16–24, 26, 28] have reported that the presence of diabetes or hypertension was related to reduced postoperative weight loss. These inconsistent findings across studies may reflect methodological differences, including variations in study design, patient populations, follow‐up duration, and definitions of both comorbidities and weight loss outcomes, as well as differences in surgical techniques, perioperative management, and adherence to postoperative recommendations. In line with previous research from the USA [16, 20], Mexico [24], and European countries such as Spain and Portugal [28], osteoarthritis did not show any association with postoperative weight loss in our study. The diagnosis of GERD in our study was not associated with postoperative weight loss, consistent with findings from other studies conducted in the USA [20, 29]. The present study found no association between the number of comorbidities and postoperative weight loss, in agreement with previous findings from Spain and Portugal [28]. In contrast, a study conducted in the USA reported that a greater comorbidity burden (seven or more conditions) was associated with reduced weight loss, while three to six comorbidities did not differ significantly from one to two comorbid conditions in terms of postoperative outcomes [20].

The interpretation of our results should take into account several limitations related to the use of routinely collected hospital data and their retrospective analysis. Although the data came from the largest center performing bariatric surgery and include a large group of typical patients, the study population may not fully represent all patients undergoing bariatric procedures. These issues should be considered when applying our findings to other settings or patient groups. Because the data come from medical records, it was not possible to fully standardize methods or diagnostic tests to diagnose chronic diseases and clinical diagnoses must have been accepted. However, all patients were treated in one department in one of the biggest obesity treatment facilities in the country, which raise the credibility that the management of each patient was consistent, and the same procedures were applied to diagnose and treat these patients. Next, in the analysis, the relationships studied were adjusted for key confounders including age, gender, and initial BMI; however, the possibility of residual confounding cannot be excluded. Due to lack of data on socioeconomic characteristics (e.g., education) in hospital database, these variables could not be included in the analysis.

Due to the incompleteness of the dataset, it was possible to analyze 41.4% of all the records for which information about weight in 1‐year follow‐up was available, which could have influenced the accuracy of estimates and further limited the possibility of generalizing the results to the population of all patients undergoing bariatric surgery in Poland. However, the distributions of age, sex, maximum body weight, or the majority of the evaluated comorbidities did not differ significantly between patients with and without follow‐up data. On the other hand, participants included in the analysis with the available body weight 1 year after surgery had slightly higher body weight and BMI at admission for the surgery and were more likely to have diabetes, hypertension, metabolic syndrome and coronary artery disease. Therefore, our findings may be more applicable to patients with a higher baseline BMI and a less favorable cardiometabolic risk profile. It is possible that a higher body mass before bariatric surgery and a slightly greater burden of cardiometabolic comorbidities served as motivating factors for better adherence to postoperative recommendations and closer cooperation with medical staff during the postoperative 1‐year period. In addition, the analysis of data for individuals with higher baseline body mass may lead to an overestimation of the average weight loss in our results.

Another limitation is the relatively short, 1‐year follow‐up period, which may not fully reflect the long‐term patterns of weight loss after bariatric surgery, especially in patients with chronic conditions. The lack of long‐term postsurgery weight data in our study is attributable to the standard institutional protocol, which provides postoperative care and monitoring only during the first year after surgery, making extension of the follow‐up period infeasible for the present analysis. This challenge is not unique to our cohort. Nurczyk et al. in their review paper draw attention to the issue of low follow‐up quality postbariatric surgeries and the need to ensure adequate data collection for better evaluation of the bariatric procedure’s outcomes [38]. In short term, we observe greater weight lost in patients that suffer from some comorbidities. However, patients lose a majority of their weight within the first 18 months after bariatric surgery [39]. After this point, they reach their nadir, so it seems that despite the limited follow‐up duration, a substantial proportion of the primary treatment effect is likely captured in our analysis. Nerveless, we acknowledge that longer observation is necessary to assess the durability of weight loss, the risk of weight regain, and long‐term health outcomes. Future prospective studies with extended than 1‐year follow‐up are recommended to evaluate the durability of weight loss, long‐term effects of bariatric surgery in this population, and overall long‐term health outcomes after bariatric surgery, particularly in patients with less common chronic diseases.

Although the method of sample selection does not allow these results to be interpreted as representative for all people undergoing bariatric surgery in Poland, still they are informative. Data of all consecutive patients from the largest bariatric surgery center in Poland were included. In the study group, there were twice as many women as men (67.05% vs 32.95%), which relates to global trends and predictions of severe obesity being more prevalent among women [40]. Large number of chronic diseases was analyzed, and multivariable analysis taking into account confounders was done. The study provides contemporary real‐world data from a large studied group reflecting current clinical practice and patient characteristics. It provides setting‐specific evidence that may complement the broader literature.

## 5. Conclusion

As the vast majority of patients undergoing bariatric surgery have at baseline other chronic diseases, the pre‐ and postoperative care should be multidisciplinary and take into account various therapeutic needs. The occurrence of comorbidities did not adversely affect postoperative weight loss. Specifically, the presence of metabolic syndrome and COPD was independently associated with greater total weight loss at 1 year. Further prospective studies designed specifically to investigate these associations may elucidate the observed mechanisms.

## Funding

No funding was received for this manuscript.

## Conflicts of Interest

The authors declare no conflicts of interest.

## Supporting Information

Additional supporting information can be found online in the Supporting Information section.

## Supporting information


**Supporting Information** Supporting Information contains four tables. Supporting table 1 presents comparison of selected traits in groups of patients with and without follow‐up data on weight. Supporting table 2 shows change in body weight in weight loss group in a 1‐year observation. Supporting table 3 shows associations between chronic diseases and total weight loss calculated for body weight at admission for the surgery (%WL)—results of multivariable linear regression. Supporting table 4 provides results on the association between selected chronic diseases and total weight loss calculated for maximum recorded body weight (%maxWL)—results of multivariable linear regression.

## Data Availability

The data that support the findings of this study are available on request from the corresponding author. The data are not publicly available due to privacy or ethical restrictions.
