# Supplementary material for: Associations Between Obesity‐Related Comorbidities and Weight Loss After Bariatric Surgery: A Retrospective Analysis
Source: J Obes. 2026 Jul 18;2026:8878737. doi: 10.1155/jobe/8878737 (PMC13379895; doi:10.1155/jobe/8878737)
Supplement: Supplementary file 1 — Supporting Information Supporting Information contains four tables. Supporting table 1 presents comparison of selected traits in groups of patients with and without follow‐up data on weight. Supporting table 2 shows change in body weight in weight loss group in a 1‐year observation. Supporting table 3 shows associations between chronic diseases and total weight loss calculated for body weight at admission for the surgery (%WL)—results of multivariable linear regression. Supporting table 4 provides results on the association between selected chronic diseases and total weight loss calculated for maximum recorded body weight (%maxWL)—results of multivariable linear regression. [file JOBE-2026-8878737-s001.docx]

**Supplementary materials**

**Supplementary table 1** Comparison of demographic and anthropometric traits in groups of participants with follow-up weight with remaining patients for whom no data on body weight after one year was available

| Variables | With follow-up weight  N=601 | Without follow-up weight  N=850 | p value  (M-W U test) |
| --- | --- | --- | --- |
| Age, median (Q1-Q3) years | 42.0 (35.0 – 51.0) | 42.8 (35.9 – 50.5) | 0.393 |
| Sex | | | |
| Male, % | 32.95 | 35.53 | 0.308 |
| Female, % | 67.05 | 64.47 |  |
| Weight before BS, median (Q1-Q3) kg | 130  (116.5-146.0) | 126  (115.0 – 144.0) | 0.033* |
| Max weight before BS, median (Q1-Q3) kg | 138.9  (120.0 – 152.0) | 139.4  (122.0-153.0) | 0.635 |
| BMI before BS, median (Q1-Q3) kg/m^2^ | 45.2  (41.5 - 49.6) | 43.94  (40.6 – 48.4) | 0.000* |
| Diabetes, % | 33.6 | 28.4 | 0.033* |
| Hypertension, % | 71.2 | 62.0 | 0.000* |
| Metabolic syndrome, % | 59.7 | 54.0 | 0.030* |
| Heart failure of any etiology, % | 8.5 | 6.0 | 0.068 |
| Coronary artery disease, % | 6.8 | 4.4 | 0.040* |
| COPD, % | 3.5 | 2.2 | 0.149 |
| Asthma, % | 6.8 | 9.3 | 0.092 |
| Varicose veins of the lower limbs, % | 20.8 | 22.8 | 0.359 |
| Osteoarthritis, % | 18.6 | 19.8 | 0.591 |
| GERD, % | 11.2 | 12.2 | 0.527 |
| Hepatic steatosis, % | 11.3 | 8.4 | 0.059 |
| Periodic back and joint pain, % | 8.7 | 10.4 | 0.280 |

**Note:** * p value <0.05; M-W U test –Mann-Whitney U test

**Abbreviations:** BMI, body-mass index; GERD, gastroesophageal reflux disease; COPD, Chronic obstructive pulmonary disease; T2DM, type 2 diabetes mellitus; WL, weight loss; EWL, excess weight loss; EBMIL, excess body-mass index loss; SG, laparoscopic sleeve gastrectomy; RYGB, laparoscopic Roux-en-Y gastric bypass; BS, bariatric surgery

**Supplementary table 2** Change in body weight in weight loss group in 1 year observation

| Variables | Mean (%) | SD |
| --- | --- | --- |
| %WL | 30.79 | 14.84 |
| %maxWL | 33.50 | 14.42 |
| %EWL admission weight | 56.50 | 26.44 |
| %EWL max weight | 59.58 | 24.79 |
| %EBMIL admission weight | 67.19 | 30.28 |
| %EBMIL max weight | 69.98 | 27.99 |

**Note:** SD, standard deviation;

**Abbreviations:** %WL- percent of the total weight loss calculated for body weight at admission for the surgery; %maxWL - percent of the total weight loss calculated for maximum recorded body weight; EWL, excess weight loss; EBMIL, excess body-mass index loss;

**Supplementary table 3** Association between chronic diseases and total weight loss calculated for body weight at admission for the surgery (%WL) – results of multivariable linear regression

| Diseases | Beta | Standard error | 95% CI | | p value |
| --- | --- | --- | --- | --- | --- |
|  |  |  | Lower | Upper |  |
| Metabolic syndrom |  |  |  |  |  |
| Model 1 ^a^ | 3.177 | 1.286 | 0.652 | 5.703 | 0.014* |
| Model 2 ^b^ | 3.686 | 1.259 | 1.214 | 6.158 | 0.004* |
| Model 3 ^c^ | 2.405 | 1.125 | 0.195 | 4.615 | 0.033* |
| Model 4 ^d^ | 2.351 | 1.140 | 0.113 | 4.590 | 0.039* |
| Hypertension |  |  |  |  |  |
| Model 1 ^a^ | 3.641 | 1.407 | 0.877 | 6.404 | 0.010* |
| Model 2 ^b^ | 2.929 | 1.468 | 0.046 | 5.812 | 0.046* |
| Model 3 ^c^ | 1.589 | 1.309 | -0.982 | 4.160 | 0.225 |
| Model 4 ^d^ | 1.544 | 1.312 | -1.033 | 4.121 | 0.240 |
| COPD |  |  |  |  |  |
| Model 1 ^a^ | 9.268 | 3.280 | 2.824 | 15.712 | 0.005* |
| Model 2 ^b^ | 7.809 | 3.112 | 1.695 | 13.923 | 0.012* |
| Model 3 ^c^ | 6.522 | 2.763 | 1.094 | 11.951 | 0.019* |
| Model 4 ^d^ | 6.464 | 2.767 | 1.028 | 11.900 | 0.020* |
| Osteoarthitis |  |  |  |  |  |
| Model 1 ^a^ | -3.788 | 1.641 | -7.012 | -0.565 | 0.021* |
| Model 2 ^b^ | -2.539 | 1.594 | -5.671 | 0.593 | 0.112 |
| Model 3 ^c^ | -2.114 | 1.414 | -4.893 | 0.664 | 0.135 |
| Model 4 ^d^ | -2.073 | 1.417 | -4.857 | 0.711 | 0.144 |
| GERD |  |  |  |  |  |
| Model 1 ^a^ | -4.288 | 2.023 | -8.262 | -0.313 | 0.035* |
| Model 2 ^b^ | -3.624 | 1.904 | -7.365 | 0.117 | 0.058 |
| Model 3 ^c^ | -2.179 | 1.696 | -5.510 | 1.152 | 0.199 |
| Model 4 ^d^ | -2.323 | 1.706 | -5.675 | 1.029 | 0.174 |
| Number of comorbidities (ref. 2&3) |  |  |  |  |  |
| 0 |  |  |  |  |  |
| Model 1 ^a^ | -4.293 | 1.986 | -8.194 | -0.392 | 0.031* |
| Model 2 ^b^ | -4.350 | 1.940 | -8.161 | -0.540 | 0.025* |
| Model 3 ^c^ | -3.100 | 1.728 | -6.494 | 0.293 | 0.073 |
| Model 4 ^d^ | -3.037 | 1.735 | -6.446 | 0.371 | 0.080 |
| 1 |  |  |  |  |  |
| Model 1 ^a^ | -3.856 | 1.891 | -7.569 | -0.142 | 0.042* |
| Model 2 ^b^ | -4.001 | 1.785 | -7.508 | -0.494 | 0.025* |
| Model 3 ^c^ | -2.803 | 1.590 | -5.927 | 0.320 | 0.079 |
| Model 4 ^d^ | -2.743 | 1.597 | -5.881 | 0.395 | 0.086 |
| 4+ |  |  |  |  |  |
| Model 1 ^a^ | -1.719 | 1.529 | -4.722 | 1.285 | 0.261 |
| Model 2 ^b^ | -1.210 | 1.502 | -4.159 | 1.740 | 0.421 |
| Model 3 ^c^ | -1.102 | 1.335 | -3.725 | 1.520 | 0.409 |
| Model 4 ^d^ | -1.178 | 1.347 | -3.825 | 1.469 | 0.382 |

**Note:** * p value <0.05;

^a^ Model 1 – crude;

^b^ Model 2 - adjusted to: age and sex;

^c^ Model 3 - adjusted to: age, sex and initial BMI;

^d^ Model 4 - adjusted to: age, sex, initial BMI and type of surgical procedure;

**Abbreviations:** COPD, chronic obstructive pulmonary disease; GERD, gastroesophageal reflux disease

**Supplementary table 4** Association between selected chronic diseases and total weight loss calculated for maximum recorded body weight (%maxWL) – results of multivariable linear regression

| Diseases | Beta | Standard error | 95% CI | | p value |
| --- | --- | --- | --- | --- | --- |
|  |  |  | Lower | Upper |  |
| Diabetes |  |  |  |  |  |
| Model 1 ^a^ | 2.740 | 1.303 | 0.181 | 5.300 | 0.036* |
| Model 2 ^b^ | 3.078 | 1.290 | 0.543 | 5.612 | 0.017* |
| Model 3 ^c^ | 2.068 | 1.145 | -0.180 | 4.320 | 0.071 |
| Model 4 ^d^ | 1.941 | 1.183 | -0.383 | 4.264 | 0.101 |
| Metabolic syndrom |  |  |  |  |  |
| Model 1 ^a^ | 3.423 | 1.248 | 0.971 | 5.876 | 0.006* |
| Model 2 ^b^ | 3.949 | 1.218 | 1.555 | 6.342 | 0.001* |
| Model 3 ^c^ | 2.389 | 1.089 | 0.249 | 4.528 | 0.029* |
| Model 4 ^d^ | 2.298 | 1.102 | 0.133 | 4.463 | 0.037* |
| Hypertension |  |  |  |  |  |
| Model 1 ^a^ | 3.810 | 1.366 | 1.126 | 6.494 | 0.005* |
| Model 2 ^b^ | 3.143 | 1.422 | 0.351 | 5.936 | 0.027* |
| Model 3 ^c^ | 1.527 | 1.266 | -0.961 | 4.014 | 0.228 |
| Model 4 ^d^ | 1.468 | 1.268 | -1.023 | 3.961 | 0.247 |
| COPD |  |  |  |  |  |
| Model 1 ^a^ | 9.064 | 3.189 | 2.799 | 15.328 | 0.005* |
| Model 2 ^b^ | 7.614 | 3.018 | 1.685 | 13.543 | 0.012* |
| Model 3 ^c^ | 6.281 | 2.669 | 1.038 | 11.524 | 0.019* |
| Model 4 ^d^ | 6.202 | 2.672 | 0.953 | 11.451 | 0.021* |
| Osteoarthitis |  |  |  |  |  |
| Model 1 ^a^ | -4.406 | 1.592 | -7.533 | -1.279 | 0.006* |
| Model 2 ^b^ | -3.214 | 1.544 | -6.246 | -0.182 | 0.038* |
| Model 3 ^c^ | -2.258 | 1.367 | -4.943 | 0.428 | 0.099 |
| Model 4 ^d^ | -2.205 | 1.369 | -4.895 | 0.485 | 0.108 |
| GERD |  |  |  |  |  |
| Model 1 ^a^ | -4.371 | 1.966 | -8.233 | -0.508 | 0.027* |
| Model 2 ^b^ | -3.714 | 1.846 | -7.340 | -0.088 | 0.045* |
| Model 3 ^c^ | -2.186 | 1.638 | -5.404 | 1.032 | 0.183 |
| Model 4 ^d^ | -2.376 | 1.648 | -5.615 | 0.862 | 0.150 |
| Number of comorbidities (ref. 2&3) |  |  |  |  |  |
| 0 |  |  |  |  |  |
| Model 1 ^a^ | -4.877 | 1.927 | -8.663 | -1.091 | 0.012* |
| Model 2 ^b^ | -5.047 | 1.877 | -8.733 | -1.360 | 0.007* |
| Model 3 ^c^ | -3.213 | 1.671 | -6.496 | 0.070 | 0.055 |
| Model 4 ^d^ | -3.127 | 1.677 | -6.422 | 0.168 | 0.063 |
| 1 |  |  |  |  |  |
| Model 1 ^a^ | -4.056 | 1.835 | -7.660 | -0.452 | 0.027* |
| Model 2 ^b^ | -4.240 | 1.727 | -7.633 | -0.848 | 0.014* |
| Model 3 ^c^ | -2.885 | 1.536 | -5.902 | 0.132 | 0.060 |
| Model 4 ^d^ | -2.801 | 1.542 | -5.830 | 0.229 | 0.070 |
| 4+ |  |  |  |  |  |
| Model 1 ^a^ | -1.498 | 1.484 | -4.413 | 1.417 | 0.313 |
| Model 2 ^b^ | -0.901 | 1.453 | -3.755 | 1.953 | 0.535 |
| Model 3 ^c^ | -0.956 | 1.289 | -3.488 | 1.575 | 0.458 |
| Model 4 ^d^ | -1.066 | 1.300 | -3.620 | 1.488 | 0.413 |

**Note:** * p value <0.05;

^a^ Model 1 - crude;

^b^ Model 2 - adjusted to: age and sex;

^c^ Model 3 - adjusted to: age, sex and initial maxBMI;

^d^ Model 4 - adjusted to: age, sex, initial maxBMI and type of surgical procedure;

**Abbreviations:** COPD, chronic obstructive pulmonary disease; GERD, gastroesophageal reflux disease
